# Bio-herbicidal potential of wheat rhizosphere bacteria on *Avena fatua* L. grass

**DOI:** 10.1080/21655979.2021.1877413

**Published:** 2021-02-08

**Authors:** Wei Li, Shuo Shen, Hongyu Chen

**Affiliations:** a Academy of Agriculture and Forestry Sciences, Qinghai University, Xining, China; b State Key Laboratory of Plateau Ecology and Agriculture, Qinghai University, Xining, China; c Scientific Observing and Experimental Station of Crop Pest in Xining, Ministry of Agriculture, Xining, China; d Key Laboratory of Agricultural Integrated Pest Management of Qinghai Province, Xining, China

**Keywords:** Bacteria, herbicidal activity, rhizosphere soil, wheat varieties

## Abstract

In order to isolated and identified the bacterial strains from wheat rhizosphere and evaluated the effect of different concentration of bacterial fermentation broth on the wild oats weed growth. This experiment carried out the separation and purification of dominant bacterial strains from the wheat rhizosphere soil, and performed the fermentation broth biological activity assessment by measured the seed germination and plant growth from 20 wheat varieties. The results had shown that the bacterial fermentation broth inhibits the growth of wild oat seedlings and plants to varying degrees, bacterial strains of X3, X4, X8, X12, X16 and X20 has certain level of inhibition activity and X20 has the highest herbicidal effectiveness. According to molecular biology identification, obtained superior bacterial strains X20 was *Bacillus* as potentially inhibitor for developing of bacterial-based bioherbicides for wild oats weed control management in the wheat field.

## Graphical abstract

**Figure:**
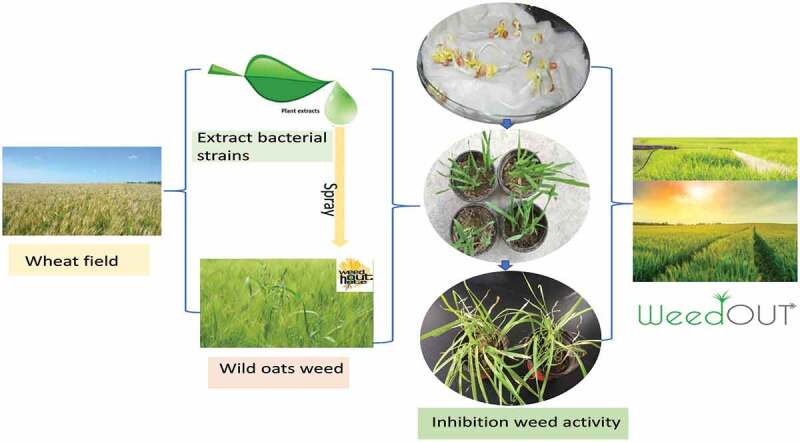

## 1. Introduction

Weeds is major limiting factor of crop growth, especially directly influence the wheat system productivity through improving management input of labor, equipment, chemical costs, and etc. At the same time, weeds indirectly affect wheat production from compete for resources, harbor pests, interfere with water management further reduce crop yield and increase processing costs [1,2]. Wild oats (*Avena fatua L*.) are the most harmful malignant weeds in the main food crops producer of wheat fields owing to the large reproduction capacity and strong adaptability as well as easily to spread and cause disasters. According to statistics reported that 75.5 million hm^2^ of farmland suffers from wild oats and losses 17.5 billion kg food among 135 million hm^2^ farmland in China. While 60% of the wheat sown area about 870 thousand ha farmland were damaged by wild oats in Qinghai province [3].

At present, commonly controlling weeds and management strategy including agricultural and chemical methods, such as mechanical/manual removal, spray chemicals while suppressed by high investment and poor durability [4–6]. Although chemical herbicides have better effectiveness such as spray of glyphosate was well control weed growth at short time, while the effectiveness will reduced when longtime applied owing to rapidly developing of weed resistance biotypes [7–9]. In addition, chemical herbicide and pesticide residues was hard to degrade and cause harmful effect on organisms and environmental from contamination of water and land as well as gases [10–12]. Fortunately, with the global attention to the substitution of inorganic agriculture, reduce the application of chemical herbicides to prevent the development of herbicide-resistant weeds, and biological agents were subsumed to weed management strategy [13,14]. Bio-herbicides has target specificity and produced from natural source that was valuable and potential developed aspect of environmental protection and food safety [15].

Rhizosphere microorganisms as the essential and active component of various ecosystems, such as play a vital role in promoted plant nutrients absorption, defended against pests and pathogens, enhanced different type of tolerate to resistance non-biological or biological stress [16–20]. Flores-Vargas isolated *Pseudomonas* has a biological control effect on winter wheat downy brome [21]. Notably the most advantage of using microbial herbicides to control weeds is the powerful targeting selectivity. Finally, applying soil rhizosphere microbes as alternative herbicide approach to control weeds can reduce production costs and the dependence of chemical herbicides as well as increase the environmental protection practically.

Therefore, objectives of this study were isolated and identified potential bacterial strains from the rhizosphere of varieties wheat, and investigated the influence of distinct concentration of bacterial fermentation broth on the wild oats weed in view of seed germination and seedling growth, aims to obtained superior bacterial strains as inhibitor for developing of bacterial-based bio-herbicides for wild oats weed control management in wheat field.

## 2. Materials and methods

### 2.1. Isolated and purification of microorganisms

The test plant wild oats (*Avena fatua L*.) seeds and microbial strains were provided by the Institute of Plant Protection Institute of Qinghai Academy of Agricultural and Forestry Sciences. Bacterial medium (LB) was made by the using of 5 g yeast powder, 10 g peptone, 10 g sodium chloride, 20 g agar, and dilute to 1000 mL, and then sterilize at 121°C for 30 min. The microbial strains isolated from the wheat rhizosphere and selected by inoculation needle, and appropriate hyphae were scribed and cultured on LB medium (placed in an incubator at 35°C for 7 day). The colonies with pure color in the medium were selected, part of hyphae was selected by the inoculation ring and inoculated in the new medium, and repeated for 2–3 times until the colonies with good growth condition were obtained.

### 2.2. Preparation of bacterial fermentation broth with different concentrations

The purified bacterial strains were inoculated into the fermentation broth (5 g yeast powder, 10 g peptone, and 10 g sodium chloride, and dilute to 1000 mL, and then sterilize at 121°C for 30 min) and incubated in a constant temperature shaker for 3d under 35°C and 200 r/min. After precipitating the cultured fermentation broth, filter the supernatant with double-layer filter paper. The supernatant was diluted with sterile water and diluted to three concentration gradients of 10, 20, and 50 times for seed germination treatment. The diluted fermentation broth medium and sterile water seed treatment was used as controls.

### 2.3. Determination of seed germination and seedling growth in petri dishes

The seeds of wild oats were soaked in 2.5% sodium hypochlorite solution with uniform size and neat shape and stirred with a magnetic stirrer for 10 min. And then washed with sterile water for 3 times (3 min each time) to wash away the residual sodium hypochlorite solution on the seeds, soak up the water by absorbent paper and soak in distilled water overnight. After two days later, wild oat seeds with consistent whiteness were selected for determination.

After take out the autoclaved glass petri dish (d = 7 cm) covered with double-layer filter paper, and add 5 ml the filtered fermentation broth of the strains with dilution concentrations of 10 times, 20 times, and 50 times into the treated petri dishes. Then, take three petri dishes and add clean water as control, and place 16 wild oat seeds with the same whiteness in each petri dish. Each concentration treatment was repeated three times and place in a constant temperature incubator at 25°C for cultivation, and keep the moisture moderate. Dark conditions were set in the first three days, and light culture was started from the fourth day. Besides, 1 h of drying was done every day, and a few drops of treatment liquid were added to the petri dish every day to ensure the filter paper was wet and avoid drought.

The seed germination rate was determined after the 3rd day of cultured. The standard of seed germination was that the radicle penetrated the seed coat by 2 mm. The bud length, root length, fresh weight and dry weight was tested on the 7th day, and the germination rate and inhibition rate of seedling were calculated.

Germination rate (%) = number of germinated weeds seeds/total number of tested weeds tested × 100%.

### 2.4. Pot experiment

Planting soil (University farm station) was used to plant 20 pots of wild oats and 3 pots as control. Put the purified strain in the culture medium, placed in shaker at 35°C and 200 r/min and cultivated for 72 h, spray the filtered strain fermentation broth with a watering can at 20 ml/pot stems and leaves once. Plants sprayed with water were used as controls.

### 2.4. Data processing

The national center for biotechnology information (NCBI) website was used for bacterial comparison, data processing, and figure drawing in Excel 2016, while variance analysis of various indicators were performed by SPSS 20.0. Sequence comparison was using the basic local alignment search tool in Gene-Bank to search for homologous DNA sequences in the DNA sequence database, and to compare the similarity of the measured DNA sequences strains to judge the similarity of species.

## 3. Results and discussion

Chemical herbicides have become a vicious circle management method due to the large amount of residues in the rice, weeds re-rampancy and the improvement of resistance. Isolated and using microbial herbicides from the natural environment carrier can reduce the dependence of chemical herbicides and protect the planting ecological environment, which has important scientific significance and application prospects. And wheat has a certain allelopathic potential through wheat living or residues to the environment release of secondary metabolites in the plant influences itself or other organisms. Therefore, this study intends to isolate and identified bacterial strains from the allelopathic wheat rhizosphere soil, and select superior strains then diluted different concentration for biological activity test to evaluate the inhibition effect by germination and seedling growth of *Avena fatua* L. grass seeds, as well as carry out the bacterial diversity analysis. It aims to provide reference for the development and utilization of microbial herbicide resources and natural environment screening.

### 3.1. Morphological characterization of plants under the application of screening strains

**Figure 1.**
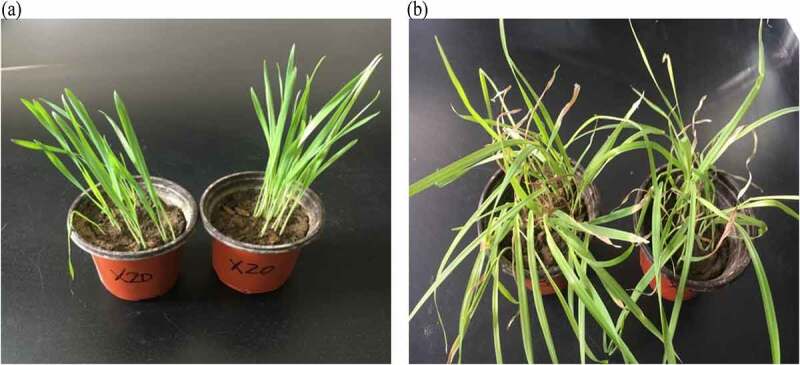
Bacterial strain X20 control (a) and experimental group (b) of wild oat plants

After the screened strain fermentation liquid was sprayed on wild oat plants, the observed plant morphological representation on 1d, 3d, 5d, and 7d showed that the wild oat plants treated with the fermentation liquid X3, X4, X8, X12, X16, and X20 showed significant differences compared with the control. Observing the plants after 7 days, it was found that the symptoms of wild oat plants leaves were water losses after spraying the fermentation broth of strains X4, X8, X16, and X20, with obviously yellow spots appeared on the stems and leaves, as well as the spiraled leaves and most of them withered yellow or even died. This phenomenon suggesting that the bacterial fermentation broth has an inhibitory effect on the growth of wild oats plants and the greater inhibitory effectiveness identified in X20 (Figure 1).

### 3.2. Effects of different concentrations of fermentation broth on the germination rate of wild oat seeds

**Figure 2.**
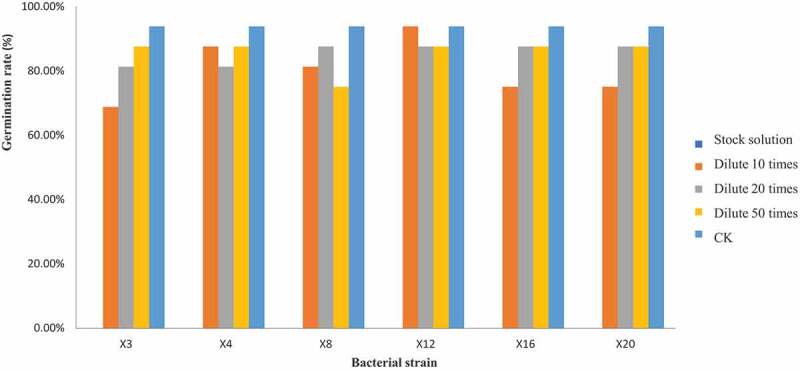
The effect of different bacterial concentration of fermentation liquor on germination inhibition rate of wild oat seeds

The fermentation broth of the bacterial strain has suppressed influence on the recipient wild oats from morphological representation, further, the influence of diluted fermentation broth on germination rate of wild oat seeds were evaluated (Figure 2). Bacterial fermentation broth could inhibit the germination and growth of wild oat seeds, and the original liquid was completely inhibiting (0%) the growth of wild oat seed seedlings. However, the germination rate of the seeds treated with the diluted fermentation broth is higher than that of the original solution. Specifically, the germination rate of diluted 10 times bacterial fermentation broth was 81.25, 93.75, 68.75, 87.50, 81.25, 81.25, 87.50, 81.25, 87.50, 93.75, 68.75, 93.75, 81.25, 75.00, 81.25, 87.50, 93.75, 100.00, 93.75, and 75.00% from X1 to X20, respectively. The lowest value appeared in X3, X11, X14 and X20 that indicating the greatest inhibitory effect. In view of germination rate under diluted 20 times bacterial fermentation broth was 93.75,87.50, 81.25, 81.25,87.50, 75.00, 93.75, 87.50, 93.75, 100.00, 75.00, 87.50, 81.25, 81.25, 81.25, 87.50, 93.75, 87.50, 93.75, and 87.50% from X1 to X20. The minimum germination rate was identified in X6, X11. While for diluted 50 times bacterial fermentation broth, the germination rate was 81.25, 81.25, 87.50, 87.50,93.75, 87.50,93.75, 75.00,87.50, 87.50, 75.00, 87.50, 87.50, 68.75, 81.25, 81.25, 81.25, 81.25, 93.75, and 87.50% from X1 to X20. The lowest germination rate was observed in X8, X11 and X14, and the germination rate of control was 93.75%. Therefore, the inhibitory effect of fermentation bacterial broth on wild oats was varied and decreased with the diluted concentration, weaker and stable inhibition effect was found at diluted 50 times. Some previous studies also similarly reported that plant extracts can effectively control the weed seeds germination and growth [22,23].

### 3.3. Effect of bacterial fermentation broth on growth characteristics of wild oats

After wild oat seeds were treated with different concentrations of bacterial fermentation broth for 7 days, the growth index of wild oat was recorded. The fermentation broth of 20 microbial strains influenced the root and bud growth of wild oat seeds. When the stock solution was diluted 10 times, the inhibitory rate of root was 59.42, 36.83, 54.24, 32.66, 61.01, 65.90, 61.15, 46.04, 60.72, 54.68, 63.31, 58.27, 58.99, 61.73, 64.17, 38.42, 51.51, 58.85, 63.74, and 40.14% from X1 to X20 (Table 1). While the root inhibitory rate value was between 25 to 40 and 10 to 30 in 20- and 50-times diluted fermentation broth. The inhibitory rate of shoot was 41.40, 11.83, 49.89, 38.28, 26.99, 36.45, 20.75, 29.14, 39.57, 37.20, 31.51, 47.20, 37.74, 43.23, 41.83, 36.02, 37.31, 34.41, 41.40, and 28.82% from X1 to X20. While the shoot inhibitory rate was between 10 to 30 and less than 10 in 20- and 50-times diluted fermentation broth (Tables 2 and Tables 3). The inhibitory rate of fresh weight was 28.00, 14.00, 29.33, 11.33, 2.00, 15.33, 8.00, 9.33, 15.33, 3.33, 10.67, 33.33, 16.67, 6.00, 13.33, 11.33, 14.67, 4.67, 10.00, and 14.00% from X1 to X20. While the fresh weight inhibitory rate was between 10 and 20 and less than 10 in 20- and 50-times diluted fermentation broth. In addition, the fermentation broth of X6, X15 and X19 strains shown strong inhibitory effect on the root growth, X3 and X12 shown strongly suppressed effect on the shoot growth and fresh weight in 10 times diluted, while no significant difference in dry weight of wild oat seedlings treated with different concentrations of fermentation broth. Notably, the average inhibitory rate of root was 54.59, 31.23, and 12.45%, shoot with 35.55, 13.34, and 7.68%, fresh weight of 13.53, 4.53, and 3.16%, dry weight of 15.45, 26.36, and 2.73% in 10, 20, and 50-times diluted bacterial fermentation broth. Therefore, to compare the indicators of shoot length, root length, fresh weight, and dry weight affection by distinct variety and concentration of bacterial fermentation broth acted on wild oat seeds, it was found that the growth of wild oat seeds were suppressed to varying degrees, obviously decreased/weaken affection was observed with the bacterial fermentation broth diluted times, and completely suppressed in bacterial stock solution.

**Table 1.** Effects of 10-times dilution of bacterial fermentation broth on seedling growth of wild oat seeds

Concentration	Strain	Root length(cm)	Shoot length(cm)	Fresh weight(g)	Dry weight(g)
Stock solution		0Bb	0Bb	0Bb	0Bb
Dilute 10 times	X1	2.82 ± 0.53Cc	5.45 ± 0.45Cc	1.08 ± 0.05Cc	0.08 ± 0.01Ac
-	X2	4.39 ± 0.46Cc	8.20 ± 0.70Aa	1.29 ± 0.61Ac	0.10 ± 0.01Dd
-	X3	3.18 ± 0.25Cc	4.66 ± 0.68Cc	1.06 ± 0.07Cc	0.08 ± 0.01Cc
-	X4	4.68 ± 0.22Cc	5.74 ± 0.21Aa	1.33 ± 0.04Cc	0.12 ± 0.02Aa
-	X5	2.71 ± 0.30Cc	6.79 ± 0.20Cc	1.47 ± 0.05Cc	0.12 ± 0.01Cc
-	X6	2.37 ± 0.17Cc	5.91 ± 0.13Cc	1.27 ± 0.06Cc	0.09 ± 0.01 Cd
-	X7	2.70 ± 0.26Cc	7.37 ± 0.18Cc	1.38 ± 0.05Cc	0.09 ± 0.01Cc
-	X8	3.75 ± 0.30Cc	6.59 ± 0.19Cc	1.36 ± 0.09Cc	0.09 ± 0.01 Cd
-	X9	2.73 ± 0.22Cc	5.62 ± 0.16Cc	1.27 ± 0.06Cc	0.09 ± 0.01Cc
-	X10	3.15 ± 0.28Cc	5.84 ± 0.17Cc	1.45 ± 0.05Cc	0.10 ± 0.01Cc
-	X11	2.55 ± 0.23Cc	6.37 ± 0.15Cc	1.34 ± 0.07Cc	0.08 ± 0.01Cc
-	X12	2.90 ± 0.23Cc	4.91 ± 0.16Cc	1.00 ± 0.08Cc	0.09 ± 0.01Cc
-	X13	2.85 ± 0.24Cc	5.79 ± 0.17Cc	1.25 ± 0.08Cc	0.10 ± 0.01Cc
-	X14	2.66 ± 0.27Cc	5.28 ± 0.14Cc	1.41 ± 0.07Cc	0.09 ± 0.01Cc
-	X15	2.49 ± 0.26Cc	5.41 ± 0.18Cc	1.30 ± 0.08Cc	0.07 ± 0.01Cc
-	X16	4.28 ± 0.56Cc	5.95 ± 0.22Cc	1.33 ± 0.06Cc	0.08 ± 0.01Aa
-	X17	3.37 ± 0.25Cc	5.83 ± 0.18Cc	1.28 ± 0.08Cc	0.11 ± 0.01Cc
-	X18 X19 X20	2.86 ± 0.22Cc 2.52 ± 0.24Cc 4.16 ± 0.25Cc	6.10 ± 0.18Cc 5.45 ± 0.16Cc 6.62 ± 0.17Cc	1.43 ± 0.06Cc 1.35 ± 0.07Cc 1.29 ± 0.06Cc	0.10 ± 0.01Cc 0.09 ± 0.01Cc 0.09 ± 0.01Cc
CK	-	6.95 ± 0.55Aa	9.30 ± 0.57Aa	1.50 ± 0.11Aa	0.11 ± 0.00Aa

CK- Control; X1 to X20-different bacterial strains extracted from the rhizosphere of wheat.

**Table 2.** Effects of 20- times dilution of bacterial fermentation broth on seedling growth of wild oat seeds

Concentration	Strain	Root length(cm)	Shoot length(cm)	Fresh weight(g)	Dry weight(g)
Stock solution		0Bb	0Bb	0Bb	0Bb
Diluted 20 times	X1	4.89 ± 0.2Dd	8.10 ± 0.23Aa	1.38 ± 0.02Dd	0.10 ± 0.01Aa
-	X2	5.10 ± 0.14Cc	9.41 ± 1.49Aa	2.00 ± 0.07Aa	0.08 ± 0.01Cc
-	X3	5.46 ± 0.40Dd	7.33 ± 0.45Aa	1.56 ± 0.07Aa	0.09 ± 0.01Cc
-	X4	6.37 ± 0.34De	8.25 ± 0.45Aa	1.43 ± 0.06Cc	0.07 ± 0.00Cc
-	X5	3.84 ± 0.11Dd	6.51 ± 0.20Cc	1.39 ± 0.02Cc	0.11 ± 0.01Cc
-	X6	3.18 ± 0.23Dd	8.09 ± 0.13Dd	1.52 ± 0.05Aa	0.07 ± 0.01Cc
-	X7	5.04 ± 0.19Dd	10.3 ± 0.62Aa	2.06 ± 0.07Aa	0.12 ± 0.01Aa
-	X8	5.11 ± 0.13Dd	6.41 ± 0.48Cc	1.32 ± 0.07Cc	0.08 ± 0.01Cc
-	X9	4.56 ± 0.14Cc	7.44 ± 0.37Cc	1.41 ± 0.05Cc	0.06 ± 0.01Cc
-	X10	5.06 ± 0.20Cc	8.28 ± 0.31Cc	1.47 ± 0.05Cc	0.07 ± 0.01Cc
-	X11	4.89 ± 0.18Cc	9.33 ± 0.45Cc	1.55 ± 0.06Cc	0.08 ± 0.01Cc
-	X12	3.62 ± 0.46Cc	8.80 ± 0.13Aa	1.40 ± 0.06Ad	0.11 ± 0.01Aa
-	X13	4.23 ± 0.23Cc	7.82 ± 0.25Cc	1.30 ± 0.05Aa	0.06 ± 0.01Cc
-	X14	3.98 ± 0.20Cc	9.47 ± 0.33Cc	1.46 ± 0.06Cc	0.08 ± 0.01Cc
-	X15	4.41 ± 0.17Cc	8.52 ± 0.30Cc	1.56 ± 0.07Cc	0.07 ± 0.01Cc
-	X16	4.91 ± 0.24Cc	5.47 ± 0.37Cc	1.40 ± 0.02Cc	0.08 ± 0.00Aa
-	X17	3.85 ± 0.19Cc	6.98 ± 0.36Cc	1.51 ± 0.04Cc	0.07 ± 0.01Cc
-	X18 X19 X20	4.33 ± 0.16Cc 5.18 ± 0.15Cc 6.48 ± 0.16Cc	7.74 ± 0.28Cc 8.03 ± 0.31Cc 9.08 ± 0.25Cc	1.48 ± 0.05Cc 1.58 ± 0.06Cc 1.39 ± 0.05Cc	0.07 ± 0.01Cc 0.08 ± 0.01Cc 0.07 ± 0.01Cc
CK	-	6.87 ± 0.55Aa	9.31 ± 0.57Aa	1.58 ± 0.11Aa	0.11 ± 0.00Aa

CK- Control; X1 to X20-different bacterial strains extracted from the rhizosphere of wheat.

**Table 3.** Effects of 50- times dilution of bacterial fermentation broth on seedling growth of wild oat seeds

Concentration	Strain	Root length(cm)	Shoot length(cm)	Fresh weight(g)	Dry weight(g)
Stock solution		0Bb	0Bb	0Bb	0Bb
Dilute 50 times	X1	4.92 ± 0.25Dd	7.56 ± 0.44Aa	1.49 ± 0.03Aa	0.09 ± 0.01Ac
-	X2	5.05 ± 0.19Cc	7.7 ± 0.43Ac	1.51 ± 0.1Aa	0.12 ± 0.01Aa
-	X3	6.03 ± 0.14Dd	8.04 ± 0.38Aa	1.53 ± 0.03Aa	0.13 ± 0.02Aa
-	X4	6.65 ± 0.44 Cd	6.75 ± 0.46Aa	1.32 ± 0.06Cc	0.08 ± 0.00Cc
-	X5	5.90 ± 0.36Ee	8.39 ± 0.39Dd	1.55 ± 0.05Ca	0.13 ± 0.01Aa
-	X6	5.88 ± 0.17Ee	8.65 ± 0.29Aa	1.42 ± 0.10 Cd	0.11 ± 0.01Aa
-	X7	7.07 ± 0.12Aa	9.62 ± 0.56Aa	1.62 ± 0.06Dd	0.12 ± 0.00Dd
-	X8	5.56 ± 0.19Dd	9.10 ± 0.14Aa	1.55 ± 0.06Aa	0.10 ± 0.01Aa
-	X9	5.23 ± 0.18Cc	8.48 ± 0.25Aa	1.47 ± 0.07Aa	0.11 ± 0.01Aa
-	X10	6.26 ± 0.15Cc	8.55 ± 0.27Cc	1.52 ± 0.08Cc	0.12 ± 0.01Cc
-	X11	5.85 ± 0.17Cc	8.96 ± 0.33Cc	1.45 ± 0.06Cc	0.10 ± 0.01Cc
-	X12	5.43 ± 0.24Dd	9.32 ± 0.42Aa	1.55 ± 0.06Aa	0.12 ± 0.01Aa
-	X13	6.34 ± 0.20Cc	9.27 ± 0.31Cc	1.58 ± 0.07Cc	0.13 ± 0.01Cc
-	X14	6.65 ± 0.18Aa	8.72 ± 0.25Aa	1.62 ± 0.06CC	0.12 ± 0.01Aa
-	X15	5.74 ± 0.16Cc	8.35 ± 0.16Cc	1.59 ± 0.07Cc	0.11 ± 0.01Cc
-	X16	5.83 ± 0.18Dd	8.83 ± 0.61Aa	1.38 ± 0.07Cc	0.10 ± 0.00Aa
-	X17	6.21 ± 0.20Cc	8.56 ± 0.45Cc	1.41 ± 0.08Aa	0.12 ± 0.01Cc
-	X18 X19 X20	7.02 ± 0.28Cc 5.99 ± 0.18Cc 6.86 ± 0.23Cc	9.14 ± 0.50Cc 8.36 ± 0.35Cc 8.82 ± 0.38Cc	1.52 ± 0.07Cc 1.40 ± 0.06Cc 1.54 ± 0.07Cc	0.13 ± 0.01Aa 0.10 ± 0.00Cc 0.12 ± 0.01Cc
CK	-	6.88 ± 0.55Aa	9.27 ± 0.57Aa	1.55 ± 0.11Aa	0.11 ± 0.00Aa

CK- Control; X1 to X20-different bacterial strains extracted from the rhizosphere of wheat.

### 3.4. Molecular identification of selected strains

**Figure 3.**
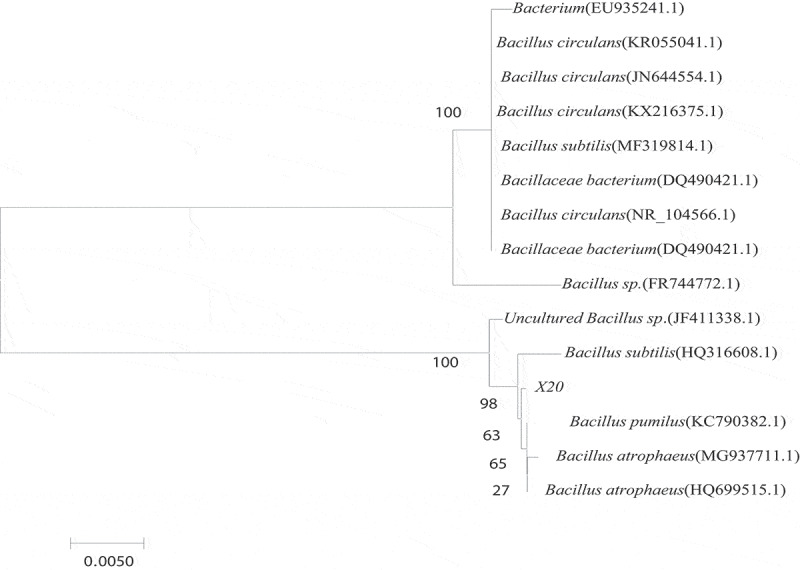
*Bacillus* 16S rRNA sequence phylogenetic tree

The 16S rRNA gene of the strain was amplified by PCR with a double-terminal common primer of 16S rDNA, and the DNA sequence was determined for homology comparison with the known sequences in National Center for Biotechnology Information- Gen-Bank. The comparison results showed that the two amplified sequences of the target sequence X20 to be tested are closest to the *Bacillus*, with more than 99% sequence similarity (Figure 3). It was determined that the strain X20 derived from the genus *Bacillus* spp., that is consistent with the fact of dominance colonization in soil bacterial owing to the ability of produce endospore and antibiotics [24,25].

The results showed that the fermentation broth of dominant bacterial isolated from wheat rhizosphere soil had a certain inhibitory effect on the seed bud length, and plant growth of wild oats. The germination stage of seeds was sensitive to the change of external environment, and the difference of the original solution and diluted was significant varied. Among them, the effect of fermentation broth of strains X4 and X8 on seed germination was greater than plant growth, indicating that the secondary metabolites produced by *Bacillus* spp. in the rhizosphere soil of wheat had herbicidal activity. In exploring different concentration gradient strains fermented liquid on wild oat seed germination and plant growth process, the influence of X20 strains fermented liquid concentrate has inhibitory effect on germination of seeds, and some of the strains fermented liquid obviously influence the plant growth. Notably, strains fermented liquid effect on seed germination is stronger than plant growth. Thus, recommend using of herbicide should be before germination duration considering the widespread weed control wild oats.

### 3.5. Description of major identified soil bacterial community from phylum to species

The bacteria isolated from wheat rhizosphere have interspecific specificity that was less harmful to cultivated plants and safe to the environment. Many researchers studied and proposed some of the rhizosphere microorganisms have herbicidal activity, such as isolated bacterial from weeds rhizosphere including *Pseudomonas, Flavobacterium, Citrobacter* and *Bacillus* showed inhibitory effects on host weeds, *Bacillales, Lysinibacillus*, and *Bacillus* were participated in plant defense [26,27]. The identified rhizosphere bacterial might be influenced the plant growth by adjust microbial activity.

**Figure 4.**
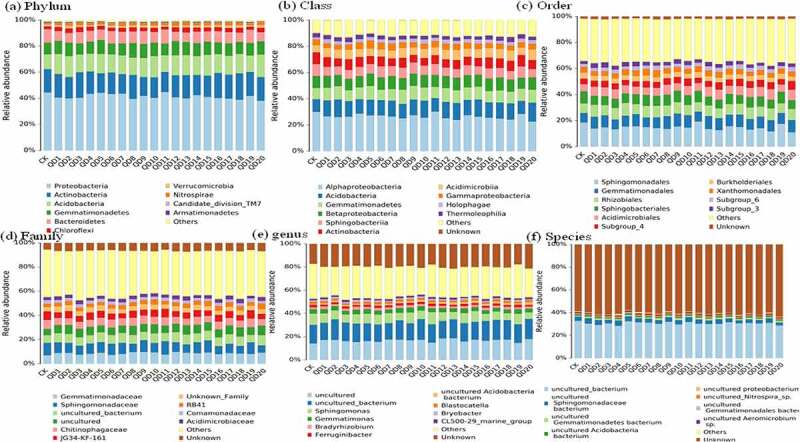
Bacterial community distribution from phylum to species

In this study, the bacterial community was predominant by *Proteobacteria* and showed richest phylum among the all variety of wheat, and lower relative abundance was found in weed exist treatments (38.1-40.6%) than control (44.5%) (Figure 4). The phylum of antibiotic producer of *Actinobacteria* was observed second enriched bacterial but RA was slightly decreased during all weed exist wheat variety (14.2–17.8%) [28,29]. *Proteobacteria* and *Actinobacteria* were known as eutrophic bacterial and contained lots of plant growth-promoting organisms [30,31]. The declined trend in weed exist treatments indicated the attenuated nutrient and resistance habitat that unfavorable to the wheat growth [32]. Whereas *Acidobacteria* as oligotrophic microbial and *Gemmatimonadetes* riches in malnourished condition and the increased relative abundance in weed exist treatments (13.4–17.4% and 9.21–10.9%) than control (11.3 and 8.7%) revealed the phenomenon of nutrient weaken due to weed competition.

Aspect of class level, the bacterial was richest in *Alphaproteobacteria* and the RA was decreased from 30.1% to 28.6–22.7% in weed exist soil, while the RA of *Acidobacteria* (9.49% to 11.7–14.2%), *Gemmatimonadetes* (4.15% to 4.78–5.82%) and *Betaproteobacteria* (9.0% to 9.0–9.9%) were increased in weed exist treatments. Additionally, *Sphingomonadales* was most predominant order during all wheat variety and the abundance was decreased from 9.1% to 6.4–8.3%, *Rhizobiales* was increased from 7.54% to 7.6–9.9%. The most proportion family of *Gemmatimonadaceae* was increased from 7.08% to 8.26–9.76% in weed exist soil. Furthermore, identify genus and species level revealed that *Uncultured_bacterium* account richest taxa and followed by *Sphingomonadaceae*. Comparison of dominant bacterial community between weed and non-weed exist wheat soil demonstrated that *Proteobacteria, Actinobacteria*, and *Acidobacteria* were most richness representatives bacterial among all treatments, whereas the RA of *Proteobacteria* and *Bacteroidetes* were declined richness in weed soil than control that revealed the habitat environment trends with declining nutrients and resistance. At present, many rhizosphere microorganisms studied lack of information on biological functions, and further test experiments should development aspect of culturing and biological function to support the microbial-based crop production.

## 4. Conclusions

The inhibitory effect of isolated bacterial fermentation broth on germination and growth of wild oat seeds, and the effectiveness was weakened gradually with dilute concentrations. The original liquid was completely suppressed (0%) the growth of wild oat seed seedlings, the inhibitory effect of fermentation bacterial broth on wild oats was decreased with the diluted concentration, the average inhibitory rate of root was 54.59, 31.23, and 12.45% in 10, 20 and 50 times diluted. Identified dominant bacterial community between weed and non-weed exist wheat soil demonstrated that *Proteobacteria* was most richness representatives bacterial among all treatments, and declined richness in weed soil from 44.5 to 38.1% indicated the attenuated nutrient and resistance habitat that unfavorable to the wheat growth. Overall, the bacteria of *Bacillus* isolated from wheat rhizosphere soil could produce secondary metabolites with herbicidal and bacteriostatic activities as well as altered bacterial community in plant growth.

## Highlights

1. *Bacillus* was identified as most suppressive effect on wild oat seedling.
2. Seeds germination was effectively inhibited in X3 (68.75%) and X20 (75%).
3. Inhibitory effect of bacterial broth on wild oats was decreased with diluted times.
4. The maximum inhibitory rate of root was 65.90% in 10 time’s diluted time.
5. Predominant *Proteobacteria* and *Actinobacteria* were reduced in weed exist wheat soil.
